# Cross-cultural adaptation and measurement properties of Youth Quality of Life Instrument-Short Form (YQOL-SF) in a developing South-East Asian country

**DOI:** 10.1371/journal.pone.0253075

**Published:** 2021-06-23

**Authors:** Men Thi Hoang, Ha Ngoc Do, Trang Quynh Dang, Hoa Thi Do, Tham Thi Nguyen, Long Hoang Nguyen, Cuong Tat Nguyen, Linh Phuong Doan, Giang Thu Vu, Toan Van Ngo, Carl A. Latkin, Roger C. M. Ho, Cyrus S. H. Ho

**Affiliations:** 1 Institute for Global Health Innovations, Duy Tan University, Da Nang, Vietnam; 2 Faculty of Medicine, Duy Tan University, Da Nang, Vietnam; 3 Youth Research Institute, Ho Chi Minh Communist Youth Union, Hanoi, Vietnam; 4 Department of Pharmaceutical Care and Health Systems, College of Pharmacy, University of Minnesota, Minneapolis, Minnesota, United States of America; 5 Institute of Health Economics and Technology, Hanoi, Vietnam; 6 Center of Excellence in Evidence-based Medicine, Nguyen Tat Thanh University, Ho Chi Minh City, Vietnam; 7 Institute for Preventive Medicine and Public Health, Hanoi Medical University, Hanoi, Vietnam; 8 Bloomberg School of Public Health, Johns Hopkins University, Baltimore, Maryland, United States of America; 9 Department of Psychological Medicine, Yong Loo Lin School of Medicine, National University of Singapore, Singapore, Singapore; 10 Institute for Health Innovation and Technology (iHealthtech), National University of Singapore, Singapore, Singapore; 11 Department of Psychological Medicine, National University Hospital, Singapore, Singapore; National Cheng Kung University College of Medicine, TAIWAN

## Abstract

This study was performed to evaluate the measurement properties of Youth Quality of Life–Short Form (YQOL-SF) in assessing the quality of life (QOL) among general youths in Vietnam. An online cross-sectional study was conducted to validate the YQOL-SF. Factor analysis (including exploratory factor analysis (EFA) and confirmatory factor analysis (CFA) was utilized to determine the factorial structure of this tool. The psychometric properties (reliability and validity) of the new factorial model were assessed. The factor analysis revealed the two-factor model of YQOL-SF including factor 1 “Belief in self and family”, and factor 2 “Environment and relationships”. Cronbach’s alpha value showed excellent internal consistency in both factors (0.911 and 0.910, respectively). Results also indicated good convergent, divergent, concurrent, and know-group validity of the two-factor model. Our study provided a promising model with different domains that were proved to be essential for the assessment of quality of life among Vietnamese youth aged 16–24. Our two-factor model affirmed that a balance between detail and length of the assessment is important to consider when selecting YQOL-SF for youths’ QOL assessment. It helped reduce the risk of redundancy and encourages high survey completion rates among participants.

## Introduction

Quality of life (QOL) is an important self-reporting indicator in assessing the development of a country, as well as the effectiveness of intervention programs for specific cohorts [1]. Unlike other traditional health outcomes such as mortality, prevalence, incidence or severity of morbidity, QOL is the self-perception of individuals regarding different life’s aspects such as physical, mental, environmental, and social components within specific cultural and social contexts [2, 3]. According to the World Health Organization, the definition of QOL is “perceptions of their position in life in the context of the culture and value systems in which they live, and in relation to their goals, expectations, standards, and concerns” [2]. Promoting QOL is an ultimate goal of many interventions with children and adolescents, particularly those with acute and chronic disabilities. In literature, QOL is closely related to individual’s health condition. Prior evidence suggests that QOL assessment was critical to detect a wide range of health problems that can influence people’s lives [4]. Furthermore, QOL is a good predictor for treatment and survival outcomes, as well as evaluating the health care disparities among healthy youths and youths with different disabilities [4, 5]. Considering QOL as an indicator of the effectiveness of intervention programs or treatment progress helps to assess the impact of these interventions on each individual’s life; thereby, enabling clinicians or policymakers to design interventions with optimal effectiveness and efficacy [6].

Each sub-population has different expectations and perceptions regarding their own QOL [6]. This difference raises the need to design and evaluate specific QOL measuring tools for different groups given that they may perceive the importance of different life aspects in different manners. In youths and adolescents, common measures of QOL such as focused only on aspects related to physiological function or ability to perform daily activities [7, 8]. Meanwhile, other factors that play an increasingly important role in the QOL of this population, such as autonomy, independence, self-confidence, ability to get along with friends, or respect from others have not been specifically mentioned [8, 9]. One potential instrument to evaluate the youth’s QOL is Youth Quality of Life Instrument (YQOL) and its short form, the Youth Quality of Life Instrument-Short Form (YQOL-SF), which has been developed and validated elsewhere [8, 9]. This instrument showed good reliability and validity in distinguishing healthy youths and those with chronic illnesses and disabilities [8, 9]. Moreover, this instrument includes elements such as sources of resilience or resources for solving problems, which are critical for youths to cope with their problems.

To date, several attempts have been made to validate the YQOL and YQOL-SF in different contexts and health conditions [8–10]. Moreover, this tool has been used in previous researches to measure QOL of children with obesity [11], or youths with obsessive-compulsive disorder [12]. However, none of the evidence was available about the psychometric properties of this instrument among Vietnamese youths. A previous study in college Filipino students attempted to evaluate their QOL by using YQOL-SF, but the authors only tested internal consistency reliability and convergent validity via identifying correlations between YQOL-SF and others instrument (e.g., mental health inventory (MHI-38) and student involvement questionnaire), while information about other validities such as concurrent validity, discriminative validity and others was not available [13]. Furthermore, although locating in the same region, the culture of Vietnam and Philippines are relatively different. While Vietnamese culture is significantly influenced by the Chinese culture [14], the Filipino culture tends to be similar in Western countries’ culture, especially Latin nations [15]. Therefore, youths in each country may perceive QOL differently, suggesting the need of different cross-cultural validation studies in each country. This paper aims to inform psychometric properties of YQOL-SF, including reliability, validity, and factor analysis in measuring QOL of Vietnamese youths.

## Method

### Study design and participants

Data of this study were derived from an online cross-sectional survey among youths aged 16–24 years old in Vietnam. Participants were included in the study if they resided in Vietnam for at least 6 months; accepted to be enrolled in the survey and provided e-informed consent. We used a snowball sampling method to recruit participants. First, a core group including people from the Youth Union in different institutions, companies, or organizations were formed. We invited them to participate and complete the online survey regarding the youths’ QOL. After survey completion, we asked them to invite other people in their network in order to complete the survey. From April to June 2020, there were 354 youths aged 16–24 living in 35 of 64 provinces of Vietnam who completed the survey. This study was approved by the institutional review board of the Youth Research Institute and performed according to the Helsinki declaration guideline [16]. Electronic informed consents were obtained from participants. For those aged below 18 years, we required agreement and confirmation from their parents/guardians to participate in the study.

### Measurement and instrument

In the current study, the Survey Monkey platform (https://www.surveymonkey.com/, San Mateo, CA, USA) was used to build the online survey. We developed a structured questionnaire to gather information about socio-demographic characteristics, health conditions, and QOL. It took 10–15 minutes to answer the questions. The questionnaire was tested and piloted among five youths to examine its appropriateness regarding language and text. Data from the pilot study were not included in the final dataset. After receiving feedback from pilot participants, we revised and uploaded the final version of the questionnaire into the online survey platform. The data collection stage was performed when we ensured that no technical problems could occur. Specific details of the questionnaire were as below:

#### Socio-demographic and health status characteristics

We collected data about age, gender, educational attainment, marital status, and living areas. We also asked participants to report whether they had any acute symptoms in the last four weeks or any chronic diseases in the last three months.

#### Youth Quality of Life-Short Form (YQOL-SF)

The YQOL-SF consists of 15 items that provide a multidimensional assessment of quality of life among youths. Psychometric data for the longer version of the scale (the Youth Quality of Life Instrument—Revised) support the reliability and validity of this instrument with acceptable Cronbach’s alpha (0.77–0.96) and reproducibility (Intra-class correlation = 0.74–0.85), good construct and known-group validities [9]. The response scale ranges from 0 = not at all to 10 = a great deal or completely. The scores are summed and then transformed to a 0 to 100 scale by using the formula below [17]:

$$transformed\;score=\frac{actual\;score-lowest\;possible\;score}{possible\;score\;range}*100$$

Where:

transformed score: the item score after being transformed

actual score: the score that patients rated for each item (range 0–10)

lowest possible score: the lowest score that patients could rate (= 0)

possible score range: the score range of each item (range 0–10)

The total score is calculated by averaging all transformed scores of 15 items. A higher score represents a higher quality of life [17]. The instrument is designed for self-administration and requires approximately 10 minutes to complete.

#### EuroQol-5 Dimensions-5 Levels (EQ-5D-5L) (6 items)

The EQ-5D-5L and EQ-Visual analogue scale (EQ-VAS], which are two measures to evaluate health-related quality of life, were used to assess the validity of the YQOL-SF [18]. These instruments were generic multi-attribute health utility instruments and among the most common scales measuring health-related quality of life and health utility. EQ-5D-5L had outstanding psychometric properties across health conditions [19]. The EQ-5D-5L evaluated participants in five dimensions: mobility, self-care, usual activity, pain/discomfort, and anxiety/depression. Each dimension had five levels of severity from 1 = “no problems”, 2 = “slight problems”, 3 = “moderate problems”, 4 = “severe problems”, and 5 = “extreme problems”. Participants selecting options 2 to 5 were classified into “Having problems” group, while other people were categorized into “No problem” group. There were 3125 health states which could be produced from the combination of options of five dimensions. Each health state can be converted to a utility index (i.e. EQ-5D index) by using the cross-walk value set for Vietnamese [20]. The EQ-VAS evaluated self-rated health condition of the participants on a 100-point scale from 0 “the worst possible” health to 100 “the best possible” health [21]. A previous systematic review concluded that EQ-5D-5L had strong correlations with physical health, pain, mental or emotional health, as well as other clinical and biological measures [19]. Thus, using the EQ-5D-5L instrument as a proxy that reflects the physical and mental health of participants is helpful to measure concurrent and known-group validities of YQOL-SF instrument.

### Statistical analysis

We performed data analysis by using STATA version 16 (Stata Corp. LP, TX, USA). Data of this study are in the S1 File. P-value < 0.05 was considered a statistical significance. Descriptive analysis was conducted including mean, standard, frequency, percentage, skewness, and kurtosis coefficients. Floor and ceiling effects were determined if the proportion of participants rating the lowest (i.e. 0 score) or highest (i.e. 10 score) score was more than 15% [22]. We also examined the reliability, factorial structure, and validity of the YQOL-SF. For reliability, Cronbach’s alpha was calculated to determine internal consistency. Cronbach’s alpha value of 0.7 or above were acceptable [23]. Other measures including domain-domain correlation, item-item correlation, item-total correlation, and Cronbach’s alpha of the domain if the item was deleted were also assessed. For factorial structure, we conducted the Exploratory Factor Analysis (EFA) to identify the structure of the YQOL-SF instrument among Vietnamese youths based on the collected data. Scree plot and parallel analysis, in combination with eigenvalues and proportion of variance explained, were utilized to detect the optimal number of factors [24]. Scree plot and parallel analysis, in combination with eigenvalues and proportion of variance explained, were utilized to detect the optimal number of factors. We kept and included items with a loading value ≥ 0.4 [24, 25].

Then, we used the Confirmation Factor Analysis (CFA) to examine the proposed factorial structure of YQOL in explaining youths’ QOL after EFA. Multiple model fit indicators with respective cut-offs were then examined to assess the model fit of observed data (with Satorra—Bentler correction for non-normality data), including [26]:

- Root Mean Square Error of Approximation (RMSEA): a value of ≤ 0.08 for good fit
- Comparative Fit Index (CFI): a value of ≥ 0.9 for acceptable fit
- Standardized Root Mean Square Residual (SRMR): a value of ≤ 0.08 for good fit

#### Validity

The factor score was calculated by averaging all transformed scores of items in each factor. A higher score indicates a higher QOL. Pearson’s correlation matrix between items-domain was computed to examine the convergent and divergent validity of the modified-YQOL-SF [27, 28]. Insufficient convergent validity was identified if the diagonal values were less than 0.4; while insufficient divergent validity was detected if the off-diagonal values at each row were higher than the diagonal values. We examined the concurrent validity of modified-QOL by calculating Spearman’s correlation matrix between two domains’ scores, EQ-5D index, and EQ-VAS. Regarding discriminant validity, we performed a t-test to compare the score of each domain between youths with and without acute symptoms in the last four weeks, chronic conditions in the last three months, having problems in mobility, self-care, usual activity, pain/discomfort, and anxiety/depression (EQ-5D domains). Cohen’s D effect size was calculated to measure the difference in factor scores between those with and without health problems. A value of 0.2 was used to identify significant differences [29].

## Results

The socio-demographic and health characteristics of the sample are presented in Table 1. The mean age of respondents was 19.2 (SD = 1.9). The majority of them were female (73.7%), had an education level above high school (71.5%), single (93.2%), and lived in urban areas (51.7%). Regarding health status, 40.7% and 15.5% of respondents suffered from acute symptoms within the last four weeks, and chronic conditions within the previous three months, respectively. Respondents self-rated high health-related quality of life (i.e., mean EQ-5D index was 0.89 and EQ-VAS was equal to 84.8).

**Table 1:** Socio-demographic and health status characteristics of respondents.

Characteristics	Male		Female		Total		p-value
	n	%	n	%	n	%	
Total	93	26.3	261	73.7	354	100.0	
Education, Above high school	60	64.5	193	74.0	253	71.5	0.084
Living location							
Urban	54	58.1	129	49.4	183	51.7	0.199
Suburban	17	18.3	39	14.9	56	15.8	
Rural	20	21.5	87	33.4	107	30.2	
Mountainous	2	2.1	6	2.3	8	2.3	
Marital status							
Single	83	89.3	245	94.6	328	93.2	0.079
Others	10	10.7	14	5.4	24	6.8	
Having acute symptoms in the last 4 weeks	32	34.4	112	42.9	144	40.7	0.152
Having chronic conditions in the last 3 months	13	14.0	42	16.1	55	15.5	0.629
**EQ-5D-5L domains**							
Having problems in mobility	27	29.0	27	10.3	54	15.3	<0.001
Having problems in self-care	17	18.3	8	3.1	25	7.1	<0.001
Having problems in usual activities	25	26.9	23	8.8	48	13.6	<0.001
Pain/Discomfort	32	34.4	66	25.3	98	27.7	0.091
Anxiety/Depression	37	39.8	120	46.0	157	44.4	0.302
	Mean	SD	Mean	SD	Mean	SD	
Age, Mean (SD)	19.0	2.2	19.3	1.8	19.2	1.9	0.345
EQ-5D index, Mean (SD)	0.82	0.31	0.91	0.13	0.89	0.20	<0.001
EQ-VAS, Mean (SD)	85.7	16.8	84.5	15.4	84.8	15.8	0.543

**Fig 1:**
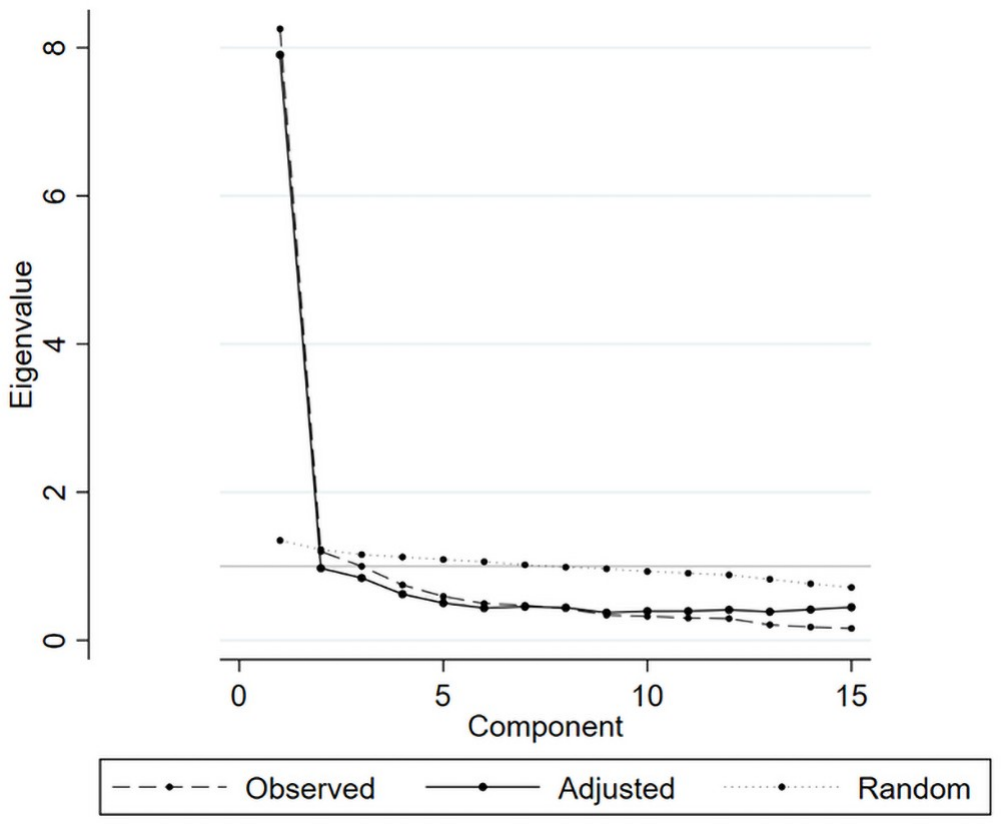
Scree parallel plot.

The scree parallel analysis and EFA identified that the 2-factor model was optimal for the YQOL assessment (Fig 1).

Table 2 revealed the results of EFA. The majority of communalities’ values were moderate. The Kaiser-Meyer-Olkin measure of sampling adequacy was 0.933 indicating that the sample was adequate for EFA. The p-value of Bartlett’s Test of Sphericity was less than 0.01 (χ2 = 4517.330; Degrees of freedom = 105; p-value <0.01) revealing that the EFA is useful for restructuring the YQOL. These factors comprised factor 1 “Belief in self and family” (7 items), and factor 2 “Environment and relationships” (7 items). Compared to the original instrument, the item Q7 “I feel alone in my life” were removed after factor analysis because the factor loading of this item was below 0.4.

**Table 2:** Exploratory factor analysis for Youth Quality of Life Instrument.

Variable	Factor 1: Belief in self and family	Factor 2: Environment and relationships	Communality
Q1 I am able to do most things as well as I want	0.453		0.5532
Q2 I feel good about myself	0.662		0.2789
Q3 I feel I am important to others	0.6003		0.4185
Q4 I am pleased with how I look	0.6497		0.3778
Q5 I feel understood by my family	0.5918		0.3842
Q6 I feel I am getting along with my family	0.6141		0.4215
Q7 I feel alone in my life*			0.9158
Q8 I am happy with the friends/colleagues I have		0.7735	0.3289
Q9 I feel I can take part in the same activities as others my age		0.7572	0.374
Q10 People my age treat me with respect		0.8143	0.2996
Q11 I feel my life is full of interesting things to do		0.7757	0.3445
Q12 I look forward to the future		0.7796	0.3592
Q13 I feel safe when I am at home		0.5544	0.6425
Q14 I feel I am getting a good education/work		0.7428	0.4172
Q15 I am satisfied with the way my life is now	0.7062		0.3106
Eigenvalue	2.56	6.01	
% Variance explained	66.8	28.4	

* Reverse-code.

Variable Q2 “I feel good about myself” had the strongest association to factor 1 latent variable, with a factor loading of 0.662, followed by Q4 “I am pleased with how I look,” Q5 “I feel understood by my family,” Q3 “I feel I am important to others,” Q15 “I am satisfied with the way my life is now,” Q6 “I feel I am getting along with my family,” and Q1 “I am able to do most things as well as I want”. Regarding factor “Environment and relationships”, Q10 “People my age treat me with respect.” was the one that had the strongest association with the latent variable, followed by Q12 “I look forward to the future”. These two factors could explain 95.2% of the total variance. Overall, our CFA results showed that the two-factor model showed more acceptable fit indices (RMSE (90%CI = 0.111 (0.100–0.122); CFI = 0.908; SRMR = 0.046; p-value = <0.001) compared to the original one-factor model (RMSE (90%CI = 0.134 (0.125–0.144); CFI = 0.842; SRMR = 0.062; p-value = <0.001).

Table 3 reveals the results of the descriptive analysis for each YQOL-SF item according to the EFA model. In the two-factor model, we excluded Q7 “I feel alone in my life”. All of 14 items had a range of scores from 1 to 10.

**Table 3:** Basic descriptions and reliability of YQOL-SF instrument.

Items	Mean	SD	Skewness	Kurtosis	Floor (%)	Ceiling (%)	Item-total correlation	Cronbach’s alpha if item deleted
**Factor 1: Belief in self and family**								
Q1	7.54	2.44	-0.94	3.26	4.2	29.9	0.718	0.908
Q2	7.52	2.47	-0.88	2.97	3.7	30.2	0.873	0.886
Q3	6.94	2.54	-0.52	2.51	4.5	22.9	0.802	0.897
Q4	7.05	2.55	-0.62	2.58	4.5	24.6	0.800	0.897
Q5	7.28	2.49	-0.79	2.90	4.0	25.7	0.826	0.893
Q6	8.00	2.37	-1.19	3.73	3.1	39.6	0.787	0.898
Q15	7.85	2.46	-1.10	3.30	2.3	38.4	0.829	0.892
**Factor 2: Environment and relationships**								
Q8	7.81	2.28	-0.98	3.37	2.3	33.9		
Q9	8.19	2.33	-1.31	3.95	2.5	45.8	0.856	0.892
Q10	8.09	2.19	-1.37	4.59	2.8	36.4	0.838	0.895
Q11	7.80	2.38	-1.01	3.30	2.8	36.4	0.863	0.891
Q12	8.39	2.02	-1.37	4.61	1.4	46.3	0.820	0.899
Q13	8.49	2.12	-1.59	5.05	1.4	50.0	0.825	0.896
Q14	8.41	2.00	-1.22	3.83	0.3	44.9	0.671	0.916
**DOMAIN SCORES**								
Factor 1: Belief in self and family	74.50	19.91	-0.65	2.98				0.910
Factor 2: Environment and relationships	81.69	17.70	-1.12	4.08				0.912

Floor and ceiling effects were determined if the proportion of participants rating the lowest (i.e. 0 score) or highest (i.e. 10 score) score was more than 15%, respectively [22]. In this analysis, 14 put of 14 items had ceiling effects, and none of the items revealed floor effects. Skewness and Kurtosis coefficients ranged from -1.59 to -0.52 and 2.51 to 5.05, respectively. Ten out of 14 items had Kurtosis coefficients above 3.0. This means that the data distribution of these items had long and fat tails as well as high and shape peaks [30]. The mean and standard deviation of item score suggested that respondents perceived positive QOL. Table 3 also depicts the reliability of the modified-YQOL-SF instrument. Internal consistency of factor 1 “Belief in self and family” and factor 2 “Environment and relationships” were excellent (Cronbach’s alpha = 0.910 and 0.912, respectively). Most of the items showed high correlation coefficients with other items in respective factors (r > 0.6).

**Fig 2:**
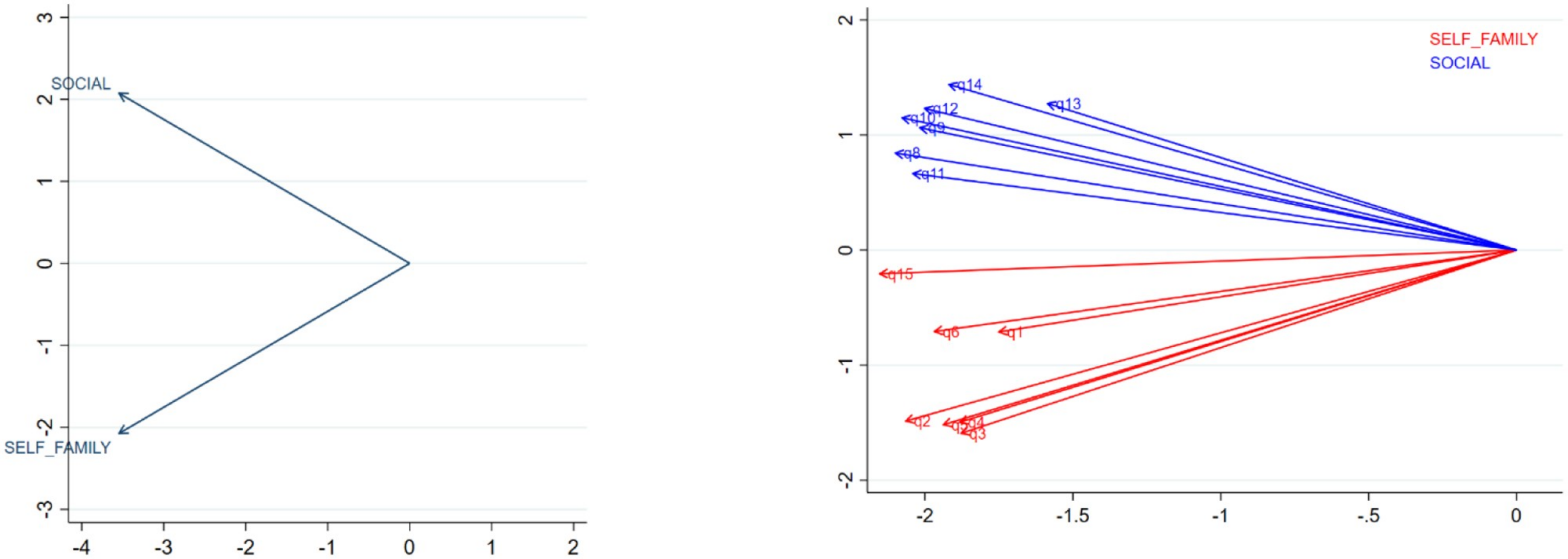
Correlation between domains (a) and items (b).

Fig 2a and 2b illustrated the factor-factor and item-item correlations. Factor 1 “Belief in self and family” was more likely to not be correlated with factor 2 “Environment and relationships”. Regarding item-item correlations, Q15 “I am satisfied with the way my life is now” in factor 1 correlated with Q11 “feel my life is full of interesting things to do” in factor 2.

Table 4 shows that all items had correlation coefficients greater than 0.4 with their respective factors’ score, indicating satisfactory convergent validity. Moreover, all items had their correlation coefficients with their factor scores greater than their correlation coefficients with other factors’ scores, implying good divergent validity.

**Table 4:** Correlation matrix between items and domain scores.

	Factor 1: Belief in self and family	Factor 2: Environment and relationships
Factor 1: Belief in self and family		
Q1 I am able to do most things as well as I want	0.616	0.586
Q2 I feel good about myself	0.818	0.655
Q3 I feel I am important to others	0.720	0.592
Q4 I am pleased with how I look	0.716	0.600
Q5 I feel understood by my family	0.754	0.606
Q6 I feel I am getting along with my family	0.707	0.663
Q15 I am satisfied with the way my life is now	0.759	0.756
Factor 2: Environment and relationships		
Q8 I am happy with the friends/colleagues I have	0.693	0.792
Q9 I feel I can take part in the same activities as others my age	0.652	0.766
Q10 People my age treat me with respect	0.669	0.805
Q11 I feel my life is full of interesting things to do	0.684	0.739
Q12 I look forward to the future	0.644	0.76
Q13 I feel safe when I am at home	0.513	0.559
Q14 I feel I am getting a good education/work	0.616	0.722

In terms of concurrent validity, Table 5 reveals the correlations between two new factors with the EQ-5D index and EQ-VAS. All correlation coefficients were statistically significant. The highest correlation coefficient was found between factor 2’s score and EQ-5D index (r = 0.4240, p<0.05), following by factor 2’s score and EQ-VAS score (r = 0.4234, p<0.05).

**Table 5:** Correlation matrix with EQ-5D index and EQ-VAS to assess concurrent validity.

	EQ-5D index	EQ-VAS	Factor 1: Belief in self and family
EQ-5D index	1		
EQ-VAS	0.4846*	1	
Factor 1: Belief in self and family	0.4069*	0.3799*	1
Factor 2: Environment and relationships	0.4240*	0.4234*	0.8030*

*p-value < 0.05.

The discriminant validity of the modified instrument was examined, and results are presented in Table 6. Overall, people having any problems, diseases, or symptoms had significantly lower scores in both factors compared to those without any problems (p<0.05).

**Table 6:** Known-group validity.

Characteristics	Factor 1: Belief in self and family			Factor 2: Environment and relationships		
	Mean (SD)	p-value	Effect size Cohen’s D (95% CI)	Mean (SD)	p-value	Effect size Cohen’s D (95% CI)
Having acute symptoms in the last 4 weeks						
No (n = 210)	77.0 (19.5)	<0.01	0.31 (0.10–0.52)	84.1 (16.5)	<0.01	0.34 (0.13–0.55)
Yes (n = 144)	70.9 (20.0)			78.2 (18.8)		
Having chronic conditions in the last 3 months						
No (n = 299)	75.7 (19.2)	<0.01	0.40 (0.11–0.69)	82.7 (16.6)	0.01	0.37 (0.08–0.66)
Yes (n = 55)	67.8 (22.6)			76.2 (22.0)		
EQ-5D-5L DOMAINS						
Having problems in mobility						
No (n = 300)	75.6 (19.5)	0.01	0.38 (0.09–0.67)	83.2 (16.9)	<0.01	0.55 (0.26–0.84)
Yes (n = 54)	68.1 (20.9)			73.6 (19.9)		
Having problems in self-care						
No (n = 410)	75.4 (19.2)	<0.01	0.63 (0.22–1.04)	82.7 (17.0)	<0.01	0.78 (0.37–1.19)
Yes (n = 25)	63.0 (25.0)			69.1 (21.7)		
Having problems in usual activities						
No (n = 306)	76.2 (19.2)	<0.01	0.65 (0.34–0.95)	83.6 (16.8)	<0.01	0.81 (0.50–1.12)
Yes (n = 48)	63.6 (21.3)			69.7 (18.6)		
Pain/Discomfort						
No (n = 256)	79.0 (17.9)	<0.01	0.87 (0.63–1.11)	85.9 (15.0)	<0.01	0.94 (0.69–1.18)
Yes (n = 98)	62.8 (20.3)			70.6 (19.5)		
Anxiety/Depression						
No (n = 197)	80.0 (18.8)	<0.01	0.65 (0.43–0.86)	86.3 (16.2)	<0.01	0.62 (0.40–0.83)
Yes (n = 157)	67.7 (19.1)			75.9 (17.8)		

## Discussion

This study informed the measurement properties of YQOL-SF in assessing QOL of the general Vietnamese youths. The instrument showed potential in measuring QOL in this sample given its good reliability and validity. Moreover, based on the factor analysis, our results showed that the two-factor model, which consisted of factor 1 “Belief in self and family” and factor 2 “Environment and relationships”, with better fit indices compared to the theoretical one-factor model. The new model was expected to be optimal and cultural-sensitive in Vietnamese youths’ QOL assessment.

In this study, participants’ scores clustered toward the high end, where the majority of the answers pointed to the score of 7, 8, 9, or 10, which represented high life satisfaction. The percentage of participants answering the lowest or highest was far above 15%, which suggested a remarkably high ceiling effect. In fact, 14 put of 14 items had ceiling effects with an average of 30% and 50% of the participants giving a score of 10 for all items in factor 1 and factor 2, respectively. This phenomenon also influenced the kurtosis coefficient as reflecting the degree of data’s outliners/peakedness [31]. Most of items with the highest ceiling effects also had the highest Kurtosis values. Our result of a high ceiling effect was in line with a previous study in the healthy sample, which was found that most of the items had a floor/ceiling effects of less than 50% [10]. This effect could be explained by the fact that we performed our study in a general youth population. It implied that future studies or interventions in disadvantaged youth population could utilize our result as the threshold to examine the success of interventions as the highest achievement in improving QOL of youths.

Regarding factor 1, our finding that the item self-esteem was highly correlated with QOL was consistent with previous studies, which showed a positive correlation between self-esteem and life satisfaction among college students [32, 33]. However, our study provided limited evidence about the contribution of self-esteem in youths’ QOL, suggesting that further studies should be warranted to examine these assumptions. In line with previous studies that showed the importance of maternal and paternal support in predicting life satisfaction of adolescent males and females [34, 35], our study highlighted the role of familial variables as critically crucial and in fact, as the second-most predictive facet of QOL in Vietnamese youth from age 16 to 24. In analyzing factor 1, it was also worthy of note that item Q7 “I feel alone in my life”—a negative self-perception intended to measure psychological health in the form of self-esteem, was dropped from our two-factor model. As previous studies had reported that the negative self-perception had been problematic in the validation of the QOL instrument among healthy adolescents [36, 37], the negatively worded items were suggested to be removed when assessing the perceived QOL of adolescents [38].

In terms of factor 2 “Environment and relationships,” our findings were in congruence with previous studies [39]. In our analysis, the correlation between observed variables and latent variables was not as strong as those in factor 1, but still, it provided meaningful insights into how positive Environment and relationships could facilitate a higher QOL in Vietnamese youth. The Q12 “I look forward to the future” could have a different interpretations which not only reflected the concept of social environment but also of self-esteem [40]. This was not to presume that the two factors in our model could be interchangeable, but it was to highlight that both factors could be associated with Q12 and both should be analyzed without one or the other being neglected when studying the youth’s future outlook. In addition to Q12, Q10 “People my age treat me with respect” was another observed variable that had a strong association with the latent variable. Previous studies found a greater reliance of adolescents on their peers in middle to late adolescence than in early adolescence [41, 42]. While our study did not stratify youths into earlier vs. later stage as in other studies, we explicitly indicated the “same age” component in our item Q10 as our education system, unlike a lot of those in Western countries, was featured to place youths of the same age in the same class and working environment.

Finally, out of all variables in factor 2, Q13 “I feel safe when I am at home” was the only one that had a correlation with the latent variable that was smaller than 0.5. We do not have an explanation for this finding, but we believed that this item was exchangeable between factor 1 and factor 2 given that the correlation coefficient of item Q13 and overall scores of these two factors were similar. Prior studies have shown that home could protect adolescents from external risk factors, while internal home environment had significant effects on adolescent health and QOL [43, 44]. The finding provides us directions to examine the role of safety within a family setting in determining the QOL among Vietnamese youth in our future research. Notably, items of factor 2 suggested that mutual understanding and respect among the youth population, especially among those of the same ages, were critically pivotal in defining the youth’s QOL. Overall, our results confirmed that factor 1 “Belief in self and family” had greater contributions to the general QOL in Vietnamese youth age 16–24 than factor 2 “Environment and relationships.” It was also important to highlight that while previous research suggested that environment played an important factor in adolescent’s health, Environment and relationships had not always been assessed [45].

The CFA result confirmed the EFA findings when showing acceptable fit indices resulted from our two-factor model. Particularly, we found that our two-factor model, which consisted of factor 1 “Belief in self and family” and factor 2 “Environment and relationships” had better fit indices compared to the theoretical one-factor model. Also, consistent with previous findings, our two-factor model delivered better fit indices when Q7 “I feel alone in my life”—a negative self-perception—was dropped from the model [36]. We named these two factors based on the items belonged to the factors as well as literature review. In the long form of YQOL-SF (i.e. YQOL-R), the development study suggested that with 41 items, the YQOL-R should be divided into four factors: self, environment, relationships and general QOL [9]. We adopted the original QOL conceptual of this instrument to name the factors including “Belief in self” (Q1, Q2, Q3, Q4, Q15), “Family” (Q5, Q6), “Environment” (Q11, Q12, Q13, Q14), and “Relationships” (Q8, Q9, Q10). Our finding was different from the study result in Philippine, where YQOL-SF was validated with the one-factor model [13]. The author did not perform EFA or CFA for exploring factorial structure of the instrument; however, when we examined one-factor model by using CFA, we found that the model fit of two-factor model was much better than the one-factor model, suggesting that the two-factor model was more appropriate for young people in Vietnam rather than the one-factor model.

In terms of reliability, the factor 1 and 2 gave equally high Cronbach alphas [0.911 and 0.910, respectively]. Our results were consistent with the majority of previous QOL studies that Cronbach alpha of the YQOL-SF was high [10, 13]. In factor 1, removing Q1 “I am able to do most things as well as I want” resulted in the highest Cronbach alpha, whereas it was Q14 “I feel I am getting a good education/work” in factor 2 being removed that resulted in the highest Cronbach alpha. Whereas most of the items in both factors showed high correlation coefficients with other items in respective factors (r > 0.6), a relatively weak correlation between Q14 and the latent variable may explain why removing Q14 improved the internal consistency in factor 2. Moreover, a wide scale of 11 in both factors allowed the data points to diverge significantly from the average value, which indicated a good measurement of variability and thus, a high internal consistency. Overall, our Cronbach alphas showed both factors in our model generate similar levels of internal consistency, and all items were useful to include in the QOL survey.

It was also noteworthy that in convergent validity, we should not expect high correlation between two instruments; instead, a moderate correlation would be preferable [46]. However, if two instruments showed high correlation, one should have the brevity or feasibility advantage to another as a reason for validation [46]. This was true in our study that both factors showed high correlation, but factor 1 “Belief in self and family” had slightly more contributions to the general QOL in Vietnamese youth age 16–24 than factor 2 “Environment and relationships.” Moreover, all items had their correlation coefficients with their factor scores greater than their correlation coefficients with other factors’ scores, implying good divergent validity, which was also consistent with previous findings [46]. In terms of discriminant validity, people having any problems, diseases, or symptoms had significantly lower scores in both factors compared to those without any problems.

Based on the results of item analysis, EFA, CFA, reliability and validity tests, we concluded that our two factor-model could be applied to the 16–24 age population in Vietnam. One of our study strengths was that we had a large sample size of total of 435 youths aged 16–24 living in 35 of 64 provinces. We also validated the psychometric properties of our survey on a large population of youths, factoring in possible confounding variables, such as illness, pain/ discomfort and anxiety/ depression. In addition, we explored the relations between our EQ-5D index with another related measure, which was EQ-VAS, through concurrent validity. There were, however, some limitations in our study. First, we did not investigate different dimensions with respect to age and gender while previous literature had shown that there were differences in QOL between adolescents (13–17 years old) and young adults (18–24) as well as males and females [47]. Our sample comprised all categories of ages from 16–24, irrespective of schooling status, which may have caused an issue of over-generalization in the study [36]. Secondly, the inclusion criteria for our sample were relatively narrow, in which participants could be qualified for the study when they lived in Vietnam for only six months. Finally, our data were collected based on self-reported information, implying possible recall and response bias.

## Conclusion

Our study provided a promising model with different domains that were proved to be essential for the assessment of quality of life among Vietnamese youth aged 16–24. For future research, our two-factor model affirmed that a balance between detail and length of the assessment is important to consider when selecting YQOL-SF for youths’ QOL assessment. It helped reduce the risk of redundancy and encourage the rate of survey completion among participants.

## Supporting information

S1 File (DTA)
